# Spatial Frequency Training Modulates Neural Face Processing: Learning Transfers from Low- to High-Level Visual Features

**DOI:** 10.3389/fnhum.2017.00001

**Published:** 2017-01-18

**Authors:** Judith C. Peters, Carlijn van den Boomen, Chantal Kemner

**Affiliations:** ^1^Department of Cognitive Neuroscience, Faculty of Psychology and Neuroscience, Maastricht UniversityMaastricht, Netherlands; ^2^Department of Neuroimaging and Neuromodeling, Netherlands Institute for Neuroscience, Institute of the Royal Netherlands Academy of Arts and Sciences (KNAW)Amsterdam, Netherlands; ^3^Department of Experimental Psychology, Helmholtz InstituteUtrecht, Netherlands; ^4^Department of Developmental Psychology, Utrecht UniversityUtrecht, Netherlands

**Keywords:** ERP, face processing, spatial frequency, learning, neuroplasticity, ASD

## Abstract

Perception of visual stimuli improves with training, but improvements are specific for trained stimuli rendering the development of generic training programs challenging. It remains unknown to which extent training of low-level visual features transfers to high-level visual perception, and whether this is accompanied by neuroplastic changes. The current event-related potential (ERP) study showed that training-induced increased sensitivity to a low-level feature, namely low spatial frequency (LSF), alters neural processing of this feature in high-level visual stimuli. Specifically, neural activity related to face processing (N170), was decreased for low (trained) but not high (untrained) SF content in faces following LSF training. These novel results suggest that: (1) SF discrimination learning transfers from simple stimuli to complex objects; and that (2) training the use of specific SF information affects neural processing of facial information. These findings may open up a new avenue to improve face recognition skills in individuals with atypical SF processing, such as in cataract or Autism Spectrum Disorder (ASD).

## Introduction

Perception of visual stimuli improves with training, but is in general highly specific for the trained stimulus set or feature. For example, learning to distinguish individuals in one set of face identities does not transfer to other face identities (e.g., Hancock et al., 2000), or across emotional expressions (Calder et al., 2000). This is generally also true for low-level features: training improves performance on a wide range of perceptual tasks (see Fine and Jacobs, 2002; Watanabe and Sasaki, 2015 for review) including discrimination of orientation (e.g., Schoups et al., 2001), texture (Karni and Sagi, 1991), coherent motion (Watanabe et al., 2001) and spatial frequency (SF; Fiorentini and Berardi, 1980), but does not transfer to other stimulus dimensions (Yu et al., 2004), stimuli (Fahle, 2004) or visual field locations (e.g., Karni and Sagi, 1991; Shiu and Pashler, 1992).

**Figure 1:**
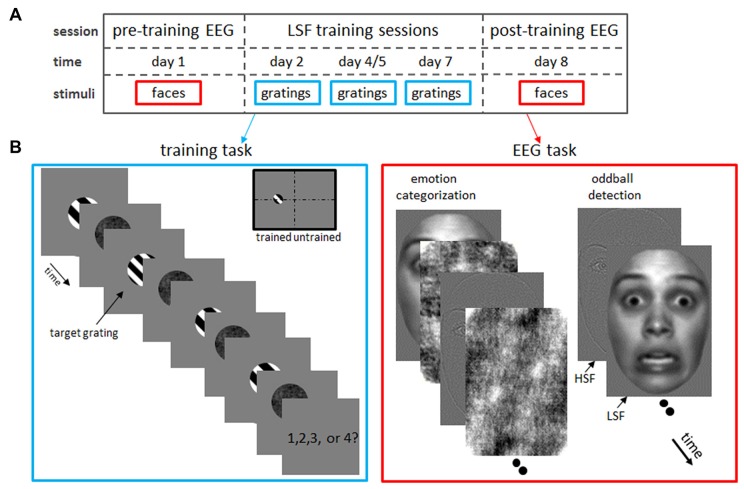
**Experimental design. (A)** Timeline of the experimental protocol. After a pre-training (“baseline”) electroencephalogram (EEG) measurement on day 1, subjects participated in behavioral sessions on day 2, 4 or 5 and 7 in which they trained low spatial frequency (LSF) discrimination on grating stimuli. Finally, a post-training EEG was acquired on day 8, while subject performed an emotion categorization and oddball detection task identical to the pre-training EEG measurements. **(B)** Tasks in the LSF training (left) and EEG (right) sessions. Left: LSF discrimination skills were trained by detecting the odd-one-out target grating which SF was increasingly similar to the reference gratings as performance improved (i.e., staircase tracking 84% accuracy). In this example trial, the grating with a different SF than the fixed SF (2 cpa) of the reference gratings, is the second grating in the row. Therefore, the correct answer is “2”. In catch trials, where two target gratings were shown, participants pressed the spacebar (instead of the number corresponding to the position of the deviant grating). Gratings were always presented in the left hemifield, to allow comparisons between trained (left hemifield) and untrained (right hemifield) visual field locations. Right: in the EEG measurements, subjects performed an emotion categorization (left image series) and oddball detection (right) task on low-pass (LSF) and high-pass filtered (HSF) faces. Note that faces were presented at the same position as the gratings in the training task (trained hemifield) or at the mirror location in the opposite hemifield (untrained hemifield).

However, it remains unknown to what extent training-induced improved sensitivity of low-level visual features (such as spatial frequency (SF), the number of black-to-white transitions in an image) transfers to complex stimuli. Here, we study whether improved sensitivity to Low SF (LSF) content, achieved by learning to discriminate black-white stripes (gratings), affects neural LSF processing in faces. LSF information in faces contains the pivotal global information necessary for proficient holistic face processing (Goffaux et al., 2005; Peters et al., 2013). In adult face perception, information carried by different SF bands is combined following a coarse-to-fine sequence (Goffaux et al., 2011; see Ruiz-Soler and Beltran, 2006 for review). LSF conveys highly important coarse information (e.g., emotional expressions) that is first extracted, before more fine-grained High SF (HSF) information is examined for further facial cues (related to for example facial age; see LSF- and HSF-filtered faces in right panel of Figure 1B).

If training LSF sensitivity indeed enhances an optimized use of information in LSF content during face processing, such an approach may lead to new skill training development to improve (emotional) face recognition abilities. Such training could aid individuals with Autism Spectrum Disorder (ASD), who have a detrimental bias towards processing information conveyed by HSF over LSF ranges, resulting in hampered recognition of faces and emotional expressions (Deruelle et al., 2004, 2008; Vlamings et al., 2010). Although there are training programs available to improve face processing skills in children with ASD (Silver and Oakes, 2001; Tanaka et al., 2003), learning a particular set of faces does not transfer easily to other face identities, expressions or general context. Moreover, face learning is a slow process (Faja et al., 2008), compared to learning of low-level visual features such as SF (e.g., Fiorentini and Berardi, 1980; Huang et al., 2008). Finally, low-level feature learning effects are long-lasting, causing a neural reorganization in the visual system (Karni and Sagi, 1993; Schoups et al., 2001), essential to training programs aiming for long-term improvements in face perception skills.

The present study investigates whether such a learning transfer is feasible. To this end, we examined four unresolved questions related to SF training:

1. Do learning effects, established by training to discriminate simple stimuli, transfer to complex objects such as faces? Therefore, we trained subjects to discriminate small LSF differences between black-white stripes (gratings) and examined whether this improved LSF sensitivity for gratings affected face processing. The extent to which learning low-level features of simple stimuli transfers to high-level object processing is (to our knowledge) yet unknown. Nevertheless, we speculate that improvements in discrimination of LSF gratings will transfer to real-world objects such as faces.
2. Does increased SF sensitivity solely transfer to processing image information in the trained SF ranges, or also to other ranges? More specifically, will LSF training transfer to low-pass (LSF) but not high-pass (HSF) filtered faces? We investigated this question by comparing neural processing of LSF and HSF faces before and after SF training. Learning-induced improvements in SF discrimination between gratings are specific for the trained SF range (Fiorentini and Berardi, 1980; Huang et al., 2008). In the same vein, we expect that training-induced LSF sensitivity will only affect processing of face images containing the trained SF band. Thus, we assume exclusive training effects for LSF- but not for HSF-faces in the present study.
3. How is such training-induced modification of LSF processing in faces reflected at the neural level? To assess neural markers of LSF learning in face perception, we recorded event-related potentials (ERP) while subjects performed the same face perception tasks before and after 3 days of LSF discrimination training (Figure 1A). The most prominent ERP component indexing face processing is the N170, a negative peak occurring at occipito-temporal sites around 170 ms post stimulus onset (Eimer, 2000). The N170 is earlier and stronger for faces containing LSF compared to HSF content (Goffaux et al., 2005; Vlamings et al., 2009; Peters et al., 2013). The neural correlates of SF discrimination training are not investigated yet, which hinders a straightforward prediction of training-related N170 modulations. Nevertheless, we hypothesize that improvements in neural LSF processing will be guided by the same neural mechanisms that attain more efficient orientation processing: orientation learning paradigms show that improved orientation discrimination results from a narrowing of the tuning-curves of orientation-selective visual neurons (Yang and Maunsell, 2004). Such a steeper slope of the tuning curve increases the neuron’s selectivity, resulting in a reduced number of responding neurons. Since ERPs reflect activity at the neural population level, narrowing of the tuning curves would hence lead to a lower ERP activity. Although exact mechanisms remain elusive, we assume that LSF discrimination learning induces a narrowing of tuning curves in SF-selective neurons, akin to the neural tuning observed in orientation learning. Therefore, we expect that neural tuning induced by LSF discrimination training will be reflected by reduced N170 responses during LSF processing in face images.
4. Is the effect of SF learning on face processing hemifield specific? Like other low-visual feature learning effects, SF improvements are specific for the trained visual field location (Fiorentini and Berardi, 1980; Huang et al., 2008). That is, even after prolonged training, SF discrimination accuracy is at pre-training level for gratings presented at untrained retinotopic locations, suggesting that learning takes places in early, retinotopically organized brain areas such as V1 (Karni and Sagi, 1993). To examine whether similar retinotopic specificities occur for training-induced alterations in face processing, LSF discrimination training was always performed in the left hemifield, whereas faces were presented in the left (trained) as well as right (untrained) hemifield. We assume that face processing is only modified for faces presented in the trained hemifield. Although we expect that the N170 is modified accordingly (i.e., only reduced N170 responses for LSF faces in the left hemifield), we would like to note that the N170 does not reflect hemifield location. That is, the N170 is generated in left and right face-selective higher-visual areas (Ghuman et al., 2014), regardless whether the faces is presented in the left or right hemifield. Therefore, the N170 might be influenced by neuroplastic changes in lower visual areas in both hemispheres, and moreover, both left- and right-hemispheric N170 activity might be affected by LSF training. The right hemispheric lateralization of face perception (Ojemann et al., 1992) might however make the training effects more pronounced for N170 responses in the right hemisphere.

In sum, we expect that LSF training will improve LSF sensitivity, leading to skilled processing of LSF (but not HSF) content in faces. At the neural level, this is reflected by a reduced N170 after training for LSF faces presented in the trained hemifield. Such a training-induced reduction is not expected for HSF faces, or LSF faces presented in the untrained hemifield. Overall, our findings confirm our expectations, suggesting the following answers to the questions raised above: (1) Improved LSF sensitivity acquired by learning to discriminate SF variations in simple stimuli (gratings) does transfer to complex objects such as faces. (2) This increased LSF sensitivity exclusively modifies processing of LSF and not HSF information in faces. (3) At the neural level, such training-induced modifications of LSF processing in faces are mirrored in reduced post-training N170 responses. (4) The observed N170 effect is specific for the trained retinotopic location (i.e., the N170 reduction only occurs for LSF faces presented in the trained hemifield).

## Materials and Methods

### Participants

Twenty healthy adults (10 males; age 18–30) with normal or corrected-to-normal visual acuity participated in two ERP measurements and three (*n* = 13) or 4 (*n* = 7) psychophysical training sessions for financial compensation or as part of their Psychology curriculum. One participant did not complete the last session and the corresponding electroencephalogram (EEG) data were therefore excluded from further analyses. This study was carried out in accordance with the recommendations of the local ethics committee of the Faculty of Psychology and Neuroscience, Maastricht University with written informed consent from all subjects. All subjects gave written informed consent in accordance with the Declaration of Helsinki.

### Experimental Procedure

Figure 1A illustrates the timeline of the experiment: on day 1, a baseline ERP measurement (“pre-training EEG”) was performed, in which the subject performed an emotion categorization task and oddball detection task on HSF and LSF faces presented in the left or right hemifield (see Figure 1B and below). Subsequently, subjects participated in 25-min sessions on day 2, 4 and 7 in which they trained LSF discrimination on grating stimuli presented in the left visual field (Figure 1B). Eight participants received the second training on day 5 instead of day 4. The SF difference between the target and reference gratings was adapted to the participant’s performance (staircase tracking 84% accuracy), resulting in improved LSF sensitivity as subjects learned to discriminate very fine varieties in LSF content. Finally, a second EEG measurement (“post-training EEG”) was carried out on day 8, in which subject performed the same tasks as in the pre-training EEG session. Task order in the EEG sessions was counterbalanced across participants.

All subjects were individually tested. They were comfortably seated in a dimly lit room shielded by a Faraday cage and monitored by cameras. Subjects were reminded throughout the sessions to maintain fixation at the middle of the screen (and limit unnecessary movements and eyeblinks during EEG recordings). Stimuli were presented at a 21″ CRT screen, (1280 × 1024 × 32 screen resolution; refresh rate 75 Hz) using the Presentation software package (v. 12.1; Neurobehavioral Systems, San Francisco, CA, USA). Subjects viewed the stimuli at a distance of 106 cm, and viewing position was stabilized using a chin-rest.

### Stimuli and Tasks

#### LSF Discrimination Training

Participants trained SF discrimination in a four Alternative Forced Choice task, in which they had to indicate which of the four sequentially presented gratings had a different SF (Fine and Jacobs, 2000; see left panel of Figure 1B). Each trial began with a 500 ms presentation of a fixation cross at the middle of a blank screen. Then, a sequence of four black-white, square-wave gratings (4.6° * 4.6° visual angle; 100% contrast) was presented at 4° eccentricity on the left horizontal meridian. Each grating had a random phase and was immediately followed by a randomly scrambled phase noise mask of the same size. Both stimuli were presented for 67 ms, followed by a 200 ms interval in which only the fixation-cross was present.

Importantly, three reference gratings had a SF of 2 cycles/degree of visual angle (cpa; reference SF), whereas the SF of the fourth grating (target grating) had a higher or lower SF (target SF), with higher or lower SF being randomly selected for each trial. This difference in SF varied across trials controlled by an adaptive staircase procedure targeting a discrimination accuracy of 84% (staircase step size 0.05% cpa; Wetherill and Levitt, 1965). The SF difference was 30% for the first trial of the first session. The starting levels for the subsequent sessions were determined based on the just noticeable difference (JND) achieved on the previous session (except for the first seven subjects who were tracked at 79, 84, 87 and 89% correct performance in session 1–4 respectively; this was corrected in the analyses by scaling obtained differences according to tracked performance). The presentation order of target and reference gratings was randomly selected for each trial. After presentation of the fourth grating and mask, a fixation cross was shown until participants responded (maximally for 1.5 s). Subjects were instructed to respond as fast and accurately as possible by pressing the keyboard keys “1” “2” “3” or “4” to indicate the target as the 1st, 2nd, 3rd or 4th grating, respectively. Participants received feedback on their response by brief (200 ms) coloring of the fixation cross (green for correct, red for incorrect or miss).

To improve task performance, we included 7% catch trials, in which two target and two reference gratings were presented. In this case, participants were required to press the spacebar. Catch trials were excluded from analyses and did not influence staircase accuracy. Note that at the beginning and throughout the session, subjects were instructed to maintain fixation at the fixation cross throughout the experiment. Training sessions lasted 25 min (400 trials) excluding self-paced breaks every 2 min.

#### EEG: Oddball Detection Task

We investigated the influence of SF training on neural processing of facial expressions using an oddball task. This orthogonal (i.e., unrelated to face perception) task ensured a continuous attention to the stimuli, yet enabled us to study face processing that occurs without any imposed task constraint that could bias facial perception. Sixty grayscale front-view photographs of Caucasian faces (50% male) with neutral (*n* = 30) or fearful (*n* = 30) expression, and four houses (odd-ball targets) served as stimuli. Face stimuli were selected from the NimStim Face Set (Tottenham et al., 2009) and subsequently trimmed to remove neck and hairline. Furthermore, all stimuli (5.4° * 3.8°) were equal in mean luminance and root mean square contrast and were presented on a homogeneous gray background of the same luminance. The SF content of each stimulus was unfiltered (broad-pass SF or BSF), or filtered with a high-pass (HSF; 6 cpa) or low-pass (LSF; 2 cpa) cut-off (see Peters et al., 2013 for details). Faces were presented at the same position as the gratings in the training task (trained hemifield) or at the mirror location in the opposite hemifield (untrained hemifield). Finally, 50 neutral faces (LSF or HSF filtered) with inverted (180° rotation) orientation were presented in each hemifield, in order to test effect of training on perception of inverted faces. All stimuli (50 trials per conditions) were in random order presented for 200 ms (Inter Stimulus Interval = 700–1100 ms) at the horizontal meridian at 4° eccentricity left or right of center. During the task, subjects were instructed to maintain fixation at the cross in the middle of the screen and press the spacebar as soon as a house was shown on the screen. Presentation of oddball trials (*n* = 32) was dispersed across the task (spacing between 11–19 stimuli). The inverted and fearful faces were presented (together with the neutral upright faces) in two separate, consecutive runs for three subjects, whereas all conditions were randomly presented in one run for all other subjects. The total task lasted about 20 min.

#### EEG: Emotion Categorization Task

The emotion categorization task employed the same stimuli as the oddball detection task, excluding inverted faces and houses. In this task, however, a scrambled version of one of the (randomly selected) face stimuli was presented immediately after the stimulus mask in order to keep stimulus processing time identical between conditions. For mask creation, phase of the face images was scrambled in the Fourier domain via random permutation, which preserves orientation content (Dakin et al., 2002). The face and mask were presented for 150 ms each, after which a fixation cross was shown 800 ms. Subjects were instructed to indicate as fast and accurately as possible whether the face had fearful or neutral emotional expression by pressing the “F” or “J”, respectively. Half of the subjects applied the reversed button order. Subsequently, participants received feedback on their response by brief (200 ms) coloring of the fixation cross (green for correct, red for incorrect, and the word “faster” for a missing response), followed by a 300 ms fixation cross. Each face was presented twice in each condition (60 trials per condition), resulting in 720 trials per session in total (360 trials per hemifield). Stimulus onset markers were not recorded in the post-training session of one subject, which missing values in the ANOVA were therefore replaced with the condition mean. The total task lasted about 20 min, including five short, self-paced breaks.

### EEG Recording

The EEG was recorded (sampling rate 500 Hz; band-pass filter of 0.01–200 Hz) from 35 AgCl scalp electrodes (extended International 10/20 system; Easycap, BrainProducts) with reference electrodes placed at the mastoids. Signals were collected using the left mastoid as reference and re-referenced off-line to the average activity of all electrodes. Horizontal and vertical electrooculograms (EOG) were recorded with bipolar electrodes placed at the external canthi and above and below the left eye. Electrode impedance was kept below 5 kOhm for all electrodes.

### Data Analysis

#### Behavioral Data

Individual performance thresholds on SF discrimination were estimated for each training day. JNDs were computed as the geometric average of the last 14 reversal points in the staircase (Wetherill and Levitt, 1965). The first session JND of one subject with insufficient reversal points was replaced by group mean JND. Improvement of LSF sensitivity was assessed by contrasting normalized JNDs of the first and third session of all subjects with a paired *t*-tested.

Reaction times of the emotion categorization task were filtered (i.e., responses below 350 ms after stimulus onset and outliers 3 standard deviations below or above condition mean were excluded) before entering the data into a repeated-measures ANOVA with SF (LSF, HSF), emotion (fear, neutral), hemifield presentation (trained, untrained stimulus position) and time (pre-training, post-training) as within-subject factors. Finally, to assess task performance, we computed d-primes indexing changes in the sensitivity of fearful facial expression detection. D-primes (*d*′) were subjected to repeated-measures ANOVA with SF (LSF, HSF), hemifield presentation (trained, untrained stimulus position) and time (pre-training, post-training) as within-subject factors. *Post hoc* paired *t*-tests were Bonferroni corrected.

#### EEG Analyses

EEG data were epoched (−200 to 900 ms, relative to stimulus onset), band-pass filtered (0.01–30 Hz; and 50 Hz Notch filter) and baseline corrected (200 ms pre-stimulus interval) using Vision Analyser (Brain Products GmbH., Munich, Germany). Artifacts from horizontal eye movements and blinks were reduced with the algorithm of Gratton et al. (1983). Trials with artifacts (i.e., samples exceeding ±75 μV, a change in voltage of 50 μV per ms, or a difference of 200 μV per 200 ms) were excluded from subsequent analyses.

For each subject-specific averaged EEG epoch of a condition, N170 peak latency and amplitude at maximal negative amplitude between 140 and 230 ms after stimulus onset were extracted for electrode PO7 (right) and PO8 (left hemisphere). In addition, mean N170 amplitudes were extracted to analyze the mean amplitude (178–182 ms) in the emotion categorization task and upward N170 slope (140–170 ms) in the oddball detection task. We opted to analyze mean amplitudes rather than subject- and condition-specific peak amplitudes to avoid averaging distortions by the trial-to-trial latency jitter in the emotion category task (e.g., Luck, 2014). Furthermore, the analysis of the upward slope was not planned* a priori*, but based on potential differences in the grand averages. Amplitudes and latencies were submitted to separate repeated-measures ANOVAs with SF (LSF, HSF), hemifield presentation (trained, untrained stimulus position) and time (pre-training, post-training) as within-subject factors. Note that we averaged across emotion (fear, neutral) for peak analyses to reduce the number of factors, since a first set of analyses did not show any interactions between emotion, SF and time. All ANOVA results were Greenhouse–Geisser corrected (but uncorrected degrees of freedom are reported) and were performed in SPSS 24 (SPSS INC, Chicago, IL, USA). Main effects and interactions that are not reported did not reach significance.

## Results

### LSF Discrimination Training

**Figure 2:**
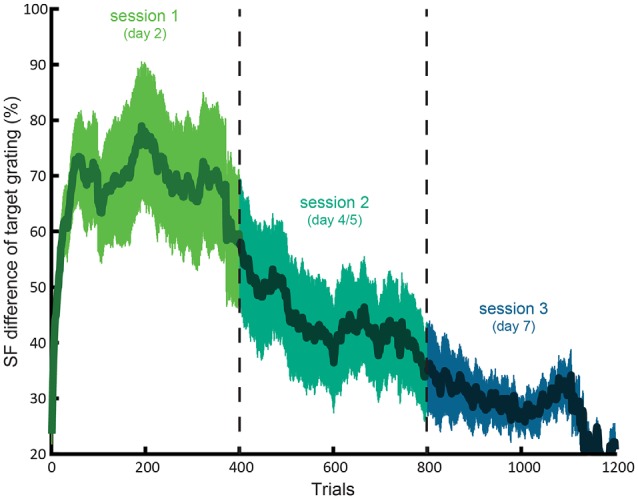
**Average learning curve for the LSF training.** The difference between the SF of the target and reference (as percentage of the SF reference grating with 2 cpa SF) as a function of concatenated trials of session 1, 2 and 3. The shaded area indicates standard error of the mean.

Participants improved LSF sensitivity across training sessions, as indicated by a lower JND in the third compared to first training session (*t*_(18)_ = 4.08; *p* = 0.0007). Figure 2 shows this gradual decrease in required SF difference across concatenated sessions.

On average, participants could detect more than twice as small differences between the SF of the target and reference gratings in the third (mean JND SF difference = 25.4%) compared to first (mean difference = 67.8%) session. The fourth session (*n* = 7) did not seem to result in further learning, as suggested by no further decrease of JND in the fourth compared to third session (*t*_(6)_ = 0.88; *p* > 0.4).

Likewise, reaction times decreased from 674 (SE = 32) in the first to 576 (SE = 40) ms in the third session (*t*_(18)_ = 2.31; *p* = 0.03). Furthermore, RTs in the third and fourth session did not differ (*t*_(6)_ = 0.95; *p* > 0.3), suggesting that learning plateaued in the fourth session.

### EEG: Oddball Detection Task

Task performance on the oddball task was excellent, with a mean accuracy of 96.8% (SE = 0.9). Accuracy did not differ between pre- and post-training (*t*_(18)_ = 0.43; *p* = 0.8). One subject had only 80% accuracy in the post-training session and was therefore excluded from subsequent analyses for this task.

**Figure 3:**
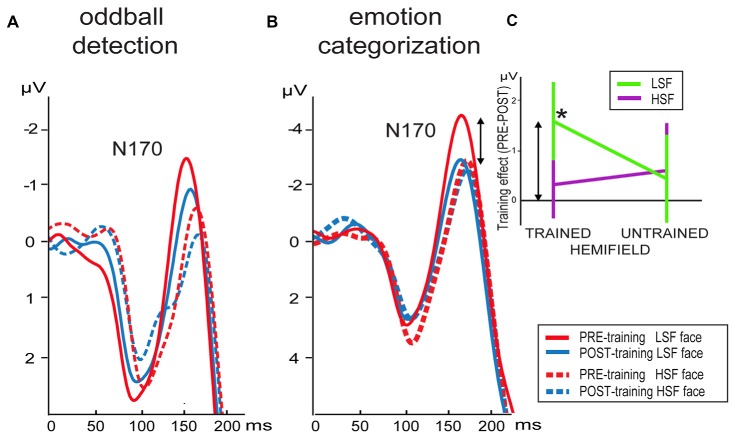
**Grand average waveforms of LSF and HSF faces presented in the trained hemifield in the pre- and post-training session elicited at electrode PO8 in the** **(A)** oddball detection and **(B)** emotion categorization task. **(C)** Differential mean N170 activation between the pre- minus post-training per hemifield stimulation (*x*-axis) and SF content in the emotion categorization task (arrows indicate the correspondence between activity shown in **B,C**). Note that the training-induced difference (*) is only present for LSF faces presented in the trained hemifield. Error bars indicate standard error of the mean.

N170 peak latencies were faster in pre- compared to post-training (*F*_(1,17)_ = 12.5, *p* = 0.003) for electrode PO7, whereas no main effects or interactions were present for peak latencies at PO8 (Figure 3A).

Analyses of N170 peak activity revealed an interaction between time and SF, which was significant for electrode PO7 (*F*_(1,17)_ = 5.3, *p* = 0.03) and a tendency at PO8 (*F*_(1,17)_ = 3.4, *p* = 0.08). *Post hoc* comparisons for PO7 did not survive Bonferroni correction. However, planned comparisons revealed the expected time*SF interaction per hemifield at electrode PO8: the N170 tended to be higher for LSF than HSF faces in the trained (left) hemifield before (*t*_(17)_ = 2.1, *p* = 0.05) but not after (*p* > 0.5) training. Such an effect was not present for stimuli presented in the untrained (right) hemifield (*p*’s > 0.1). Notably, this differential learning effect between LSF and HSF faces presented in the trained hemifield is already present in the upward slope of the N170 at PO8: mean activity was higher for LSF than HSF faces in the trained hemifield before (*t*_(17)_ = 2.3, *p* = 0.03) but not after (*p* > 0.3) training. Such an effect was not present for stimuli presented in the untrained hemifield (*p*’s > 0.2).

### EEG: Emotion Categorization Task

Participants performed the task fast (632.9 ms; SE = 19.7 ms) and accurately (mean accuracy of 74.7%; SE = 2.6%; mean *d*′ = 1.6; SE = 0.14). Reaction times revealed an interaction between emotional expression and SF (*F*_(1,18)_ = 13.0, *p* = 0.02). The only *post hoc* comparison that survived Bonferroni correction revealed that LSF neutral faces were on average 16 ms faster recognized than HSF neutral faces (*t*_(18)_ = 3.0; *p* = 0.08).

No main effects or interactions were observed for *d*′: although the training-induced increase in sensitivity was twice as high for LSF compared to HSF faces (*d*′ post- minus pre-training difference for LSF = 0.19 for HSF = 0.09), variance was too high to obtain significant differences.

As illustrated by Figure 3B, N170 peak latency at PO8 was shorter for LSF faces compared to HSF faces (*F*_(1,18)_ = 4.6, *p* = 0.046). Furthermore, stimuli presented in the trained hemifield were faster processed than stimuli in the untrained hemifield (*F*_(1,18)_ = 64.3, *p* < 0.001). However, SF, time and hemifield showed no interactions. In contrast, peak latencies at PO7 were faster for left (trained) compared to right hemifield (*F*_(1,18)_ = 84.4, *p* < 0.001), but this effect did not interact with time, nor were any other effects observed.

For electrode PO8, mean activity in the N170 window was affected by hemifield (*F*_(1,18)_ = 25.0, *p* = 0.0001), SF (*F*_(1,18)_ = 17.1, *p* = 0.001) and interactions between hemifield and SF (*F*_(1,18)_ = 5.6, *p* = 0.03), time, hemifield and SF (*F*_(1,18)_ = 7.3, *p* = 0.015) and a tendency for an interaction between time and SF (*F*_(1,18)_ = 4.1, *p* = 0.06). To interpret the two- and three-way interactions, we performed additional ANOVAs per hemifield with SF and time as factors. Results showed that ERPs elicited by stimuli presented in the untrained hemifield were affected by SF content of the face image (*F*_(1,18)_ = 17.7, *p* = 0.01), but no effects of time. In contrast, stimulus presentation in the trained hemifield was influenced by an interaction between SF and time (*F*_(1,18)_ = 10.8, *p* = 0.04): compared to pre-training, mean activity in the post-training was reduced for LSF faces (*t*_(18)_ = 2.2; *p* = 0.045) but not for HSF faces (*t*_(18)_ = 0.5; *p* > 0.5). In sum, results showed that LSF training reduced neural activity in the N170 window, but only for stimuli with *LSF content* presented in the *trained hemifield*. This selective influence of training is reflected in Figure 3C showing mean differential activity as a function of hemifield presentation and SF content.

For mean N170 activity at electrode PO7, we observed an interaction between hemifield and SF (*F*_(1,18)_ = 9.7, *p* = 0.06). *Post hoc* tests revealed that activity in the untrained hemifield was smaller for LSF than HSF faces (*t*_(18)_ = 3.7; *p* = 0.002), whereas activity did not differ in the trained hemifield (*t*_(18)_ = 0.7; *p* > 0.1).

In sum, the N170 in the right hemisphere was reduced in the post-training compared to pre-training session. Notably, this decreased processing was only observed for faces containing LSF information. Moreover, this learning effect was not only specific for trained SF, but also for trained location: the learning-related decrease in LSF processing was only present when faces were presented at the same location as the gratings in the LSF discrimination training sessions. In contrast, no differences between pre- and post-training ERP were observed for LSF faces presented in the untrained hemifield.

## Discussion

For fast and proficient face processing, facial cues conveyed by information in the LSF range are essential (Goffaux et al., 2005, 2011). Improving LSF processing might therefore increase face processing abilities. Our results showed that training-induced improvement in LSF discrimination of low-level stimuli indeed transfers to LSF processing in faces, which is accompanied by enduring changes at the neural level.

Participants learned to discriminate increasingly small SF variations in LSF gratings in a discrimination task. After only three training sessions (25 min., 400 trials), the JND between target and reference SF dropped from ~68% to ~25%, indicating a fast and strong increase in LSF sensitivity. Interestingly, this improvement in LSF perception was neurally reflected by a decrease in N170 amplitude. This reduction was exclusively observed for *LSF* faces in the *trained* hemifield in the post-training emotion categorization task. This is in line with psychophysical observations that SF learning is restricted to trained SF range and retinotoptic location (Fiorentini and Berardi, 1981). Similar learning-specific effects were also present in an oddball task, yet less pronounced. This discrepancy could result from several differences between the categorization and oddball detection task. Fast and accurate categorization of emotional expressions required more intensive processing than the passive perception in the oddball task, which could underlie the more pronounced expression of LSF training effects. That is, whereas LSF content is important for proficient, configural processing in general, it is known to play an even more pivotal role in assessing emotional expressions (Vlamings et al., 2009). Moreover, the categorization task put a high demand on attentional resources, since emotional expressions had to be correctly identified within 150 ms. This higher demand on attention resources might have contributed to lateralization towards the right hemisphere in the categorization task (Heilman and Van Den Abell, 1980), compared to the more distributed effects in the easy oddball task. Increased attentional processing might have also boosted neural face (LSF-) processing in the categorization task. The higher amplitude of the N170 in the categorization compared to the detection task (Figure 3), despite being elicited by identical face stimuli, corroborates the idea that attention differences might play a role in the observed task differences.

To our knowledge, there are only a few studies that investigated perceptual learning with EEG and none of them studied the transfer of learning effects to other stimulus categories. The majority of studies reported a decreased occipital N1 across sessions of training in line discrimination (Song et al., 2002; Qu et al., 2010). More complex results were observed in a visual texture segmentation task, showing that learning-related Visual Evoked Potential decreased for stimulus configurations where global and local orientations conflicted, but not for conflict-free configurations (Casco et al., 2004). Our reduced early neurophysiological activity is in agreement with previous findings using electrophysiology (Yang and Maunsell, 2004) and fMRI (Zhang et al., 2010) suggesting that learning narrows the tuning-curves of feature-selective visual neurons. A steeper slope of the tuning curve changes the neuron’s discrimination threshold, resulting in a sparser response (i.e., reduced number of responding neurons) at the neural population level, culminating in reduced ERPs. Such a tuning mechanism is a likely candidate to explain the reduced LSF processing in face images, resulting from the improved LSF sensitivity induced by LSF discrimination training.

Peak latencies and amplitudes were differently affected. In line with previous results (Goffaux et al., 2005; Flevaris et al., 2008; Peters et al., 2013), we observed earlier N170 peaks for LSF compared to HSF faces. This effect cannot be driven by other low-level stimulus differences, as contrast and luminance were equalized between LSF and HSF faces. Rather, this effect indicates that facial information in LSF ranges is processed faster than those in HSF ranges. Neuroimaging studies in adults suggest that LSF content in faces is not only processed faster, but also processed via different neural pathways than HSF content (Vuilleumier et al., 2003; Rotshtein et al., 2007). That is, LSF information travels via the middle occipital gyrus to an area in the fusiform gyrus specialized in face processing (the so-called fusiform face area; Kanwisher et al., 1997), where it converges with HSF information coming from inferior occipital and temporal areas (Rotshtein et al., 2007). This differential processing might be a continuation from the distinct magno- and parvocellular pathways running from retina to early visual areas which are specialized for processing coarse and fine details respectively (e.g., De Valois et al., 1982; Hess, 2004). Peak latency was not influenced by LSF training, corroborating previous findings suggesting that learning-related adaptations are reflected in reduced visual activity rather than faster processing in visual cortex (Song et al., 2002; Casco et al., 2004).

In sum, our results show—to our knowledge for the first time—that training effects based on an orthogonal task using low-level stimuli, transfer to a higher-level object processing task. That is, the training employed a fundamentally different stimulus type (gratings) and task (LSF discrimination) than the experiment in which we observed the transfer effects (face images in an oddball detection and emotion categorization task). The present study only investigated face perception, but LSF training effects might transfer to other stimuli as well. Although adequate LSF processing is particularly important for holistic face perception, it might aid configural object processing in general. Similar to our face perception expertise, acquired expertise on other object classes appears to be guided by holistic processing (e.g., Richler et al., 2011) and proficient use of LSF information (Viggiano et al., 2006). Further research could investigate whether the current LSF learning paradigm may transfer to other object classes for which configural processing is important.

Interestingly, SF learning affected neural face processing after training was finished, suggesting that training effects caused a long-lasting neural reorganization. These findings can have important implications for treatment of atypical vision. Various conditions, such as ASD (Deruelle et al., 2004, 2008; Vlamings et al., 2010), pervasive developmental disorder (Boeschoten et al., 2007) and cataract (Ellemberg et al., 1999) are associated with deteriorated HSF and/or LSF processing. Our results suggest that the neural LSF and HSF processing pathways in such individuals can be optimized by SF discrimination training, resulting in improved processing of the SF ranges that convey the most important information (e.g., LSF content in faces). SF (and orientation) decomposition is a fundamental step in vision, affecting all further visual processing stages. Improving such a cardinal aspect of vision could constitute a highly generic training approach that might complement existing specific face training programs in promoting face processing skills in atypical development.

## Author Contributions

JP and CK conceived and designed the experiments. JP and CB: data acquisition and analyses. JP, CB and CK wrote the manuscript.

## Conflict of Interest Statement

The authors declare that the research was conducted in the absence of any commercial or financial relationships that could be construed as a potential conflict of interest.
